# 

**Published:** 1904-01

**Authors:** 


THE TEXAS STATE MEDICAL ASSOCIATION will meet in Austin the fourth Tuesday in April (26th), and hold four days. It is expected that this will be the largest meeting of that body ever held, as, under the new constitution a thorough organization of the rank and file of the profession has been effected. Dr. T. J. Bennett, Austin, chairman committee of arrangements, is putting the little pot in the big one, and making other arrangements for a banquet or a reception, or both, in addition to the scientific features of the meeting.

